# Multiple Pulmonary Artery Pseudoaneurysms Secondary to Metastatic Uterine Leiomyosarcoma

**DOI:** 10.5334/jbsr.2561

**Published:** 2021-09-23

**Authors:** Teetawat Jearsirikul, Surajai Junnhu, Nantaka Kiranantawat

**Affiliations:** 1 Prince of Songkla University, TH

**Keywords:** Pulmonary arterial pseudoaneurysm, metastasis, leiomyosarcoma, sarcoma, pulmonary metasasis

## Abstract

**Teaching point**: The probable mechanism of pseudoaneurysm formation related to metastatic neoplasm is a tumor embolus penetrating and destroying the vessel wall.

Pulmonary artery pseudoaneurysm (PAP) related to metastatic neoplasm is rare. We describe a unique case of multiple PAPs secondary to metastatic uterine leiomyosarcoma and demonstrate the serial chest computed tomography to support the theory that the tumor begins as a tumor embolus, followed by infiltration and breakdown of the vessel wall, leading to aneurysmal dilatation and invading the perivascular tissue.

## Case Report

**Figure 1:**
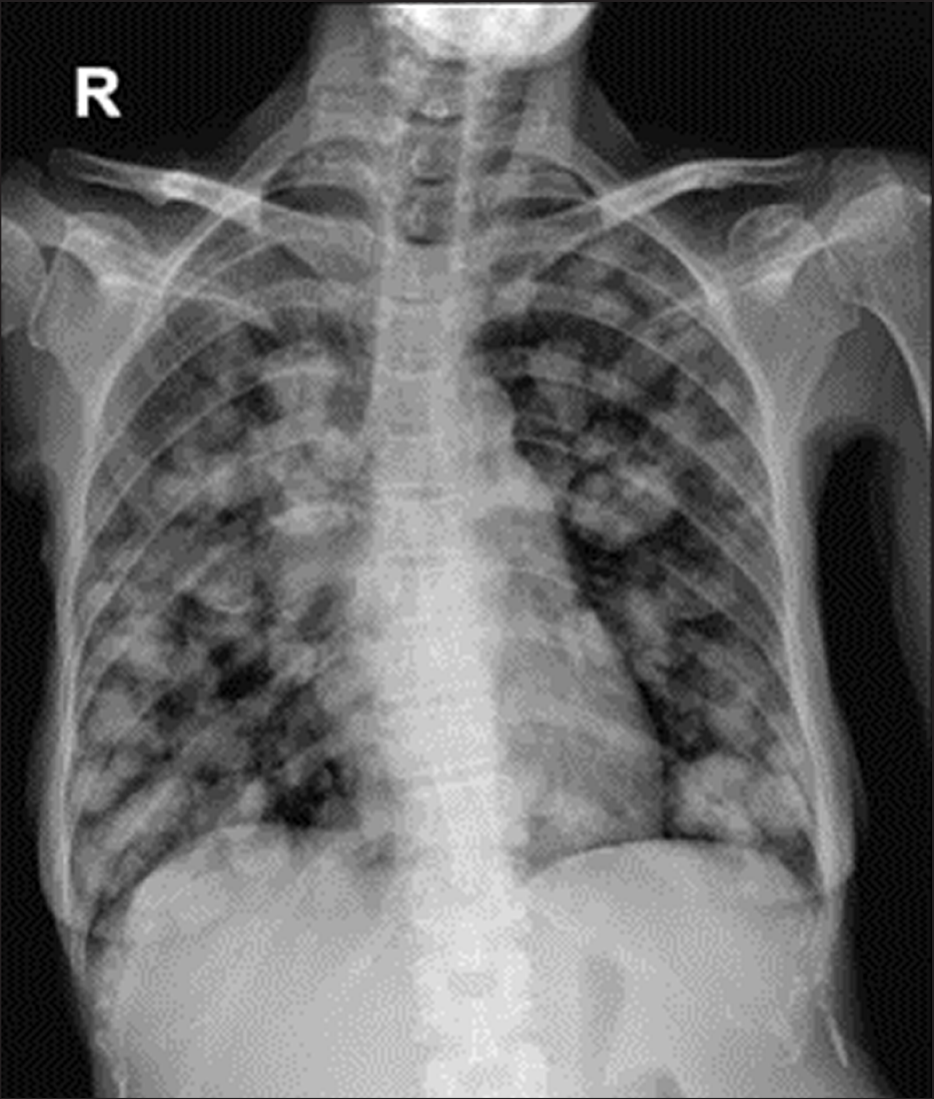
Chest radiograph showing multiple, round, variably sized masses scattering in both lungs, compatible with pulmonary metastasis.

**Figure 2:**
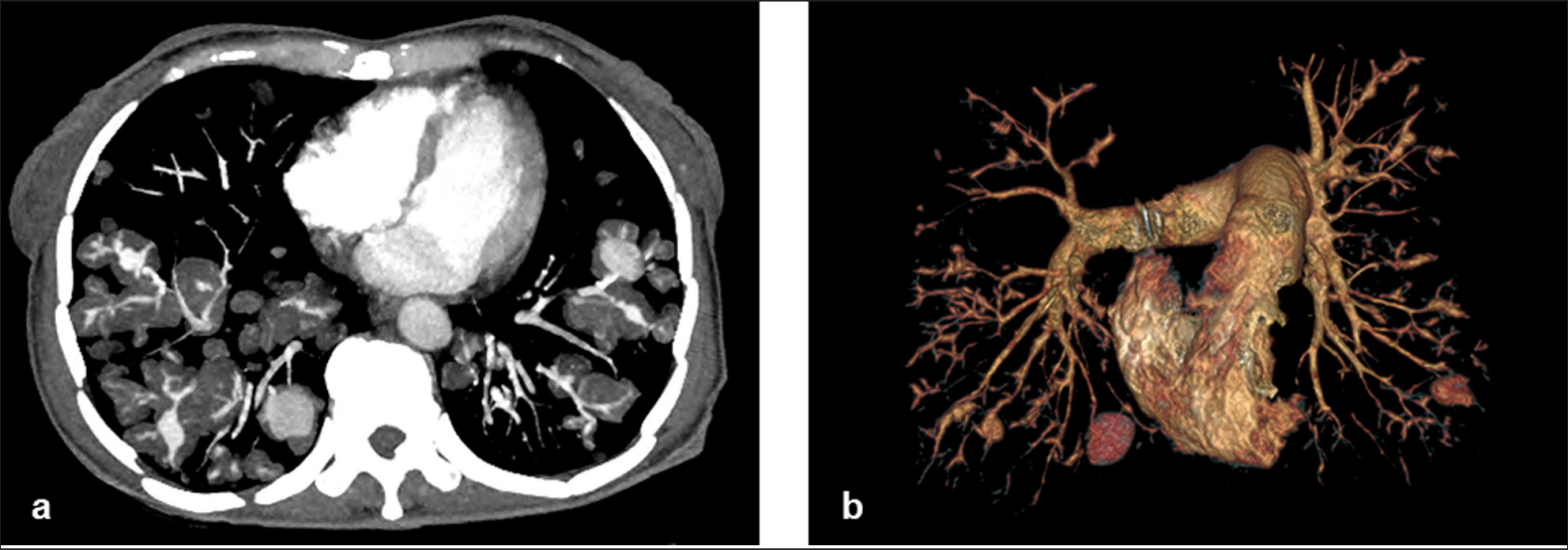
(**a**) Axial MIP image showing multiple masses, scattering in both lungs and surrounding segmental/subsegmental branches of pulmonary arteries, and multiple PAPs. (**b**) Coronal 3D Volume Rendered image showing multiple PAPs scattering in both lungs. The two largest ones are located in both lower lobes.

**Figure 3:**
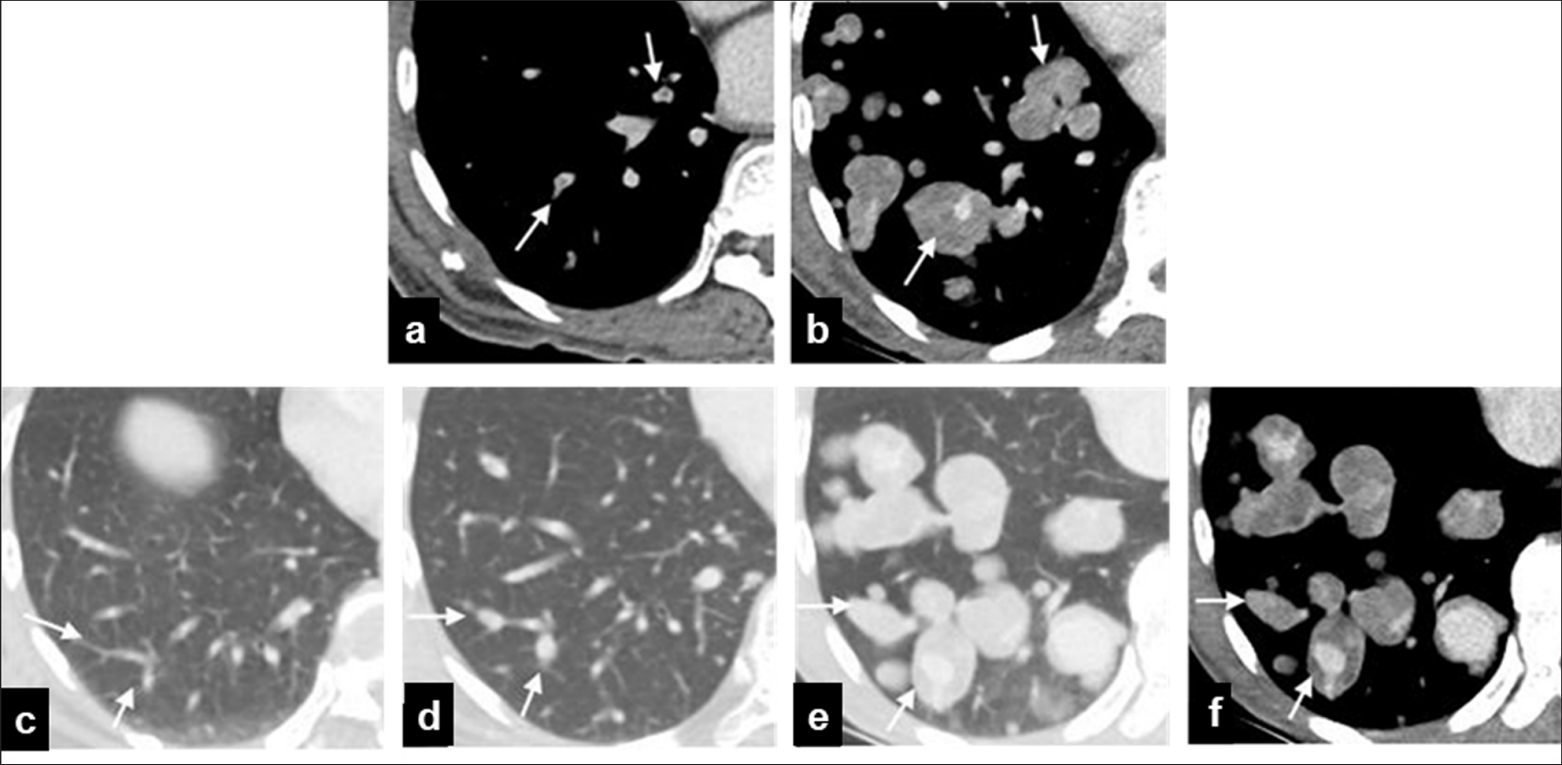
**(a)** and **(b)** Serial axial chest CT within one month showing aneurysmal dilatation and developing soft tissue masses surrounding the previously seen tumor emboli in the peripheral branches of right pulmonary arteries (arrows). (**c–f**) Serial axial chest CT at eight months prior (c), one month prior (**d**), and the present study (**e**) and (**f**) showing gradual dilatation of peripheral branches of right pulmonary arteries, and finally pseudoaneurysms with surrounding soft tissue masses (arrows).

A 49-year-old female with known uterine leiomyosarcoma and lung metastasis with multiple tumor thrombi in pulmonary arteries was receiving palliative chemotherapy. Prior to her fourth chemotherapy session, the patient developed acute-onset dyspnea. Chest radiograph revealed a larger size of the multiple well-defined pulmonary masses scattered in both lungs; therefore, progressive pulmonary metastasis was suspected (***Figure 1***). Computed tomography pulmonary angiography (CTPA) was performed to rule out acute pulmonary thromboembolism and showed innumerable heterogeneously enhanced masses scattered in both lungs, surrounding segmental branches of pulmonary arteries. Multiple fusiform pulmonary artery pseudoaneurysms surrounded by soft tissue masses were also noted (***Figure 2***). A review of serial chest computed tomography (CT) revealed tumor emboli in the peripheral branches of pulmonary arteries (***Figure 3a***), which finally developed into pseudoaneurysms with surrounding soft tissue masses (***Figure 3b***–***3f***).

## Discussion

PAP is defined as a focal dilatation of the pulmonary artery that does not comprise all three layers of the vessel wall. PAP itself is a rare condition, usually arising secondary to infection. Malignancy is a rare cause of PAP with a predilection towards primary cancer of the lungs [1]. From previous literature, we found that PAP was related to metastatic neoplasm in five cases, including metastatic sarcoma, synovial sarcoma of the right atrium, angiosarcoma of soft tissue in the neck, atrial myxoma, and breast cancer [2,3,4,5,6]. Only two cases revealed multiple PAPs: metastatic sarcoma and atrial myxoma [2,6].

The mechanism of PAP secondary to metastatic neoplasm could be explained by the “metastasize and infiltrate” theory that described the formation of cerebral artery aneurysms due to metastatic cardiac myxoma [7]. The theory proposes that the process begins as a tumor embolus, followed by infiltration and occupation of the subintimal layer of vessel walls, causing loss of the normal vessel integrity, and lastly forming a pseudoaneurysm. This theory was also supported histologically by two case reports of an intracerebral pseudoaneurysm, and multiple bilateral PAPs due to cardiac myxoma [5,8]. The authors also demonstrated tumor cells invading through the vessel walls into surrounding alveolar tissue [5].

A similar mechanism of pathogenesis is thought to have occurred in this case. From the serial chest CT, the PAPs with surrounding soft tissue masses had previously shown intraluminal thrombus and dilated pulmonary artery, which resembled tumor thrombus (***Figure 3a*** and ***3b***). The serial chest CT also showed gradual dilatation of peripheral branches of right pulmonary arteries (***Figure 3c***–***3f***); this could be due to tumor growth and continued destruction of vessel walls.

Abnormal dilatation of the pulmonary arteries and veins was also observed in the patient’s CTPA with similar findings previously described in a reported case of metastatic uterine sarcoma [9]. The only difference is that there was a clear connection between the dilated vessels with the pulmonary circulation in this patient, unlike the reported case in which the dilated vessel occurred in isolation from the pulmonary circulation, thought to have been formed by the mass itself.

In conclusion, PAPs caused by metastatic neoplasm may firstly begin as tumor emboli, later progressing into pseudoaneurysms as we elucidated in the serial chest CTs and CTPA findings in this case.
